# Examining the role of government in shaping disability inclusiveness around COVID-19: a framework analysis of Australian guidelines

**DOI:** 10.1186/s12939-021-01506-2

**Published:** 2021-07-16

**Authors:** David Colon-Cabrera, Shivika Sharma, Narelle Warren, Dikaios Sakellariou

**Affiliations:** 1 School of Social Sciences, Monash University, Level 4, 20 Chancellors Walk (Menzies Building), Clayton Campus, VIC 3800 Clayton, Australia; 2 School of Healthcare Sciences, Cardiff University, Eastgate House, Newport Road 35-43, CF24 0AB Cardiff, United Kingdom

**Keywords:** Australia, Convention on the Rights of Persons with Disabilities, COVID-19, Disability, Inclusiveness, Policy analysis

## Abstract

**Background:**

The COVID-19 pandemic has uncovered the ways in which disabled people are made more vulnerable due to structural inequalities. These vulnerabilities are the result of the interaction between individual and structural factors that shape how risk is experienced by disabled people. In Australia, these vulnerabilities are influenced by the way disability services and care for disabled people are delivered through a consumer-directed approach. We analysed the policies and documentation made by the Australian Government and state and territory governments during the pandemic to explore whether these were disability-inclusive. We aimed to unpack how these policies shaped disabled people as vulnerable citizens.

**Methods:**

Guided by documentary research, we used framework analysis to examine the policies of the Australian Government and state and territory governments. We analysed legislation that was given royal assent by the federal, state and territory governments, and documents (reports, fact sheets, guidance documents, etc.) published by the federal government and the state of Victoria (given that this state experienced the brunt of the epidemic in Australia) between February 2020 to August of 2020.

**Results:**

We found that most of the resources were not aimed at disabled people, but at carers and workers within disability services. In addition, most policies formulated by the Australian Government were related to the expansion of welfare services and the creation of economic stimulus schemes. However, while the stimulus included unemployed people, the expansion of benefits explicitly excluded disabled people who were not employed. Most of the legislation and documents offered accessibility options, though most of these options were only available in English. Disability oriented agencies offered more extensive accessibility options.

**Conclusions:**

The findings indicate a large number of documents addressing the needs of disabled people. However, disability-inclusiveness appeared to be inconsistent and not fully considered, leaving disabled people exposed to greater risk of COVID-19. Neoliberal policies in the health and welfare sector in Australia have led to an individualisation of the responsibility to remain healthy and a reliance on people as independent consumers. Governments need to take a clear stance towards the emergence of such a discourse that actively disvalues disabled people.

**Supplementary Information:**

The online version contains supplementary material available at 10.1186/s12939-021-01506-2.

## Introduction

Disabled people are disproportionately affected by the COVID-19 pandemic; existing inequalities have exacerbated, and new ones have emerged [1, 2]. Disabled people are seen as vulnerable to COVID-19 infection due to their bodily conditions, a perception which places responsibility for the prevention from infection onto the individual [3]. Research and advocacy have highlighted that, far from being an ‘inherent’ artefact of disability, this vulnerability is an artefact of interactions between structural factors and the individual. The many ways in which vulnerability is created for people with disability was documented in the October 2020 interim report of the Australian Royal Commission into Violence, Abuse, Neglect and Exploitation of People with Disability [4]. This major public inquiry highlighted that vulnerability is not a product of disability in and of itself, but results from contemporary and historical Australian Government policies, produced at Federal and State (or Territory) levels.

Insights into the ‘structural vulnerability’ of people with disability can be found in governmental half- and non-existent measures of disability inclusion. Many of these pre-date COVID-19, and reflect historical and contemporary ableist assumptions [5–7]. For example, Kavanagh and colleagues’ [8] exploration of healthcare provision for disabled people during the pandemic demonstrated that pre-existing factors were intensified: health workforce issues, limited accessibility of health-related information and health promotion programs, unequal access to health services and interventions, and limited coordination of healthcare across sectors. There is strong evidence to document the impact of COVID-19 in simultaneously exacerbating these effects while making them more evident [9–13].

Data from other countries has provided empirical insights into the specific effects of policy, including lockdowns or movement restrictions, on the experiences of people with disability. Epstein et al. [2] found that people with disability experienced barriers in accessing their usual health and social care, as well as for COVID-19-related care (including testing, also see [9–11]). Ableist medical rationing was a significant concern [2]; not only did disabled people feel discriminated when they sought care, but they were fearful of the effects of this should they require hospitalisation [2]. In this and other similar studies, people with disabilities also reported anxiety and fear around the requirement placed upon them to ‘shield’ from COVID-19 infection, as this was not always possible [1, 11, 12]. While these themes speak to health and care, people with disabilities reported significant disruptions in their everyday lives, from accessing groceries and medication to having opportunities for social connection with others [2, 11]. These effects are intensified by intersectional disadvantages [9, 13].

Across the world, governments, communities and collectives have designed varying strategies to support disability inclusion in formal responses to the pandemic. Examining disability inclusion and COVID-19 in four South American countries, Sakellariou et al. [14] found that recommendations were often made, without specific attention given to the translation of these into practice. Notably, they found that where government responses did not fully address disability inclusion, they compounded the disadvantage experienced by people living with disability. Uneven levels of inclusion of people with disability in pandemic-specific policies, detailed by Kavanagh et al. [8], range from the failure to consider disabled people as an ‘at risk’ or ‘vulnerable’ population group in pandemic management plans [3, 8] to the release of disability-specific advice (including operational plans) for people in response to community-based advocacy. In Australia, where over 4 million people, or 18 % of the population, live with disability, including 51 % of the population aged over 65 [15], both the Federal and the state governments designed and implemented policies to control the pandemic.

In this article, we focus on Australia, a country which has successfully suppressed COVID infections, to unpack the place of COVID-related policies in shaping disabled people as vulnerable citizens and thus reinforcing ideas of deficiency and personal responsibility. These constructions act in opposition to key elements of the CRPD, to which Australia is signatory, as we outline below. Our analysis is informed by the ‘Health in All Policies’ (HiAP) approach, which calls for a collaborative and multisector public policy orientation within government to simultaneously consider the health implications of their initiatives, decisions and settings, with the goal of improving health for the whole population [16, 17]. As implemented in South Australia, a HiAP focus considers health as constituted by “social, economic, political, cultural and environmental determinants” [17]. Thus, it looks beyond the individual bodies to attend to questions of equity – and the barriers to health equity – and enhanced accountability [18–20]. Below, we consider the implications for disabled people within the pandemic response, and consider how a HiAP approach might reshape their inclusion.

## COVID-19 and disability inclusion in Australia

Australia recorded its first case of COVID-19 in January 2020 [21]. Rapid increases in infection rates were subsequently reported, from 98 cases identified Australia-wide on February 28, to a cumulative total of 6058 cases by March 31, 2020 [22]. To control the spread of infection, a collaborative emergency response was formulated across all levels of government, including the establishment of the National Cabinet, an intergovernmental forum [23] to develop a cohesive national strategy for managing COVID-19 infection. Subsequently, country-wide lockdown restrictions (announced on March 21, 2020) and border closures were implemented.^1^

The strategies that were implemented to contain COVID-19 risk had specific implications for disabled people, many of which had not been considered in the rapid and decisive response to contain the pandemic. A ‘Statement of Concern,’ issued by the Royal Commission into Violence, Abuse, Neglect and Exploitation of People with Disability [25]^2^ on March 26, 2020, outlined to the government and community their responsibilities to disabled people under the Convention for the Rights of People with Disabilities (CRPD). Some of those responsibilities were discharged by the National Disability Insurance Scheme (NDIS), a consumer-directed funding model for health and social supports, and the disabled people and workers engaged through the NDIS [25].

The very nature of the NDIS has exposed disabled people to increased risk, revealing the failures of an understanding of people as autonomous consumers: the combination of a large number of carers moving between different homes with no centralised form of organisation has two implications. First, they can act to spread infection and second, it is difficult to issue directives and policies that can reach such an expansive and disparate work body. Within our methodological framework (outlined below), this aligns directly with the theme of ‘protection of people living in residential settings’ but also has implications for questions of ‘reasonable accommodations’.

Furthermore, being based on a gig economy structure, NDIS offers no incentives to carers to stay at home if they have suspected COVID as they only get paid for services they render [26]. However, not all disabled people have access to NDIS [8] as it relies on age- and eligibility-based factors; further, people need to have sufficient ‘evidence’ of their disability and its impacts to be able to access the scheme.^3^ Those who do not have access to the NDIS may be eligible for social welfare through the Disability Support Pension (DSP), although this also subject to stringent eligibility criteria.^4^ The DSP provides a basic level of support for disabled people: subject to an income and assets ‘means test’, recipients can receive a fortnightly maximum of $868.30 for single people or $654.50 if they are in a relationship. However, it provided evidence of ableist welfare policies during the COVID-19 pandemic (as we discuss in our findings below), so that people on the DSP were excluded from accessing the same financial support as those receiving other forms of social welfare. This differential support evidences the inclusion of ‘financial support’ within our methodological framework.

These factors together reimpose vulnerability onto disabled people. In attempting to address the policy and structural contributions to the experiences of increased insecurity, anxiety and precarity experienced by disabled people as a result of government and community responses to the pandemic, the Royal Commission into Violence, Abuse, Neglect and Exploitation of People with Disability [25] published a ‘Statement of Concern.’ This outlined key elements for a disability-inclusive government response to COVID-19, as shown in Table 1. Such strategies are essential to address the needs of disabled people, and to respond to, reduce and mitigate their concerns.

**Table 1:** Key elements for disability-inclusive response to COVID-19, and alignment with our methodological framework

Key element	Methodological theme
Recognition of the intersectional risks faced by particular populations of disabled people, including First Nations people	Needs of disabled people with multiple exclusions Access to education
Ensuring provision of and access to health care, essential support services, basic food and nutrition for all disabled people	Access to healthcare
Availability of public health advice and information from national health authorities - including announcements and broadcasts - through a range of accessible formats (including sign language, digital technologies, captioning, relay services, text messages, easy-to-read formats, and plain language communications)	Accessible information Reasonable accommodations for disabled people
Inclusion of disabled people in financial security protection measures, and supporting workplaces to provide reasonable adjustments for disabled people	Financial support
Implementation of specific measures to support and protect disabled people living in residential care settings	Protection of people living in residential settings

## Methods

The aim of the study was to investigate how the Australian government’s response to the COVID-19 pandemic addressed the needs, particular risks, and vulnerabilities of disabled people. The study design was guided by documentary research [27]. We used framework analysis [28] to examine legislation and other documents related to the states and Australian Government’s response to the pandemic. We aimed to collect sources published and updated from February 1st to August 31st, 2020.

### Data sources

The documents collected are divided into two major categories: the first category of documents is *legislation* (Acts and Statutory Rules) passed by states and the Australian Governments. The second category we designated as *other documents*, which included a variety of documents composed of publications, media releases, guidelines, and webpages published online by both state and federal governments.

#### Legislation

We looked at all the legislation that was given royal assent during the time period and that had any mention of COVID-19 (we excluded Bills, which were still under consideration and had not been given royal assent). We undertook a search strategy by searching in the Federal Register of Legislation which is a government website that contains the full text of the Commonwealth’s legislation. In addition, we also searched each Australian state government’s website that provided the full text of the state’s legislation including Bills considered by Parliament, Acts of Parliament, and statutory rules. We searched for legislation with the keywords COVID, COVID-19, and coronavirus with Boolean operators when available in the respective site.

#### Other documents

This category included press releases, reports, fact sheets, websites/webpages, presentations, and other types of publications. This was deemed to be necessary because the Acts of parliament as well as state regulations passed were broad in scope, and in the case of the state of Victoria, allowed for emergency powers to the Premier and the state government. This resulted in policy documents and guidelines being produced through press releases and governmental websites that delineated the mitigation strategies of the state government without having to pass legislation for every action taken. This also meant that updates to guidelines and policies were made rather quickly, and new directives were announced and implemented during the course of the pandemic.

We also noticed that while we analysed documents that were obtained from government sources, there were other resources from organisations and non-governmental bodies that were not analysed. In contrast to the legislation analysed which encompassed Australian States and Territories, as well as the Australian Government, we focused the other documents section on those produced by the Australian Government and the Victoria State Government. We focused on the state of Victoria because of its unique situation: at the time of the collection of the documents and legislation, Victoria was undergoing a second outbreak of cases that at its height saw 687 new daily cases on August 4th 2020 as recorded by the State of Victoria Department of Health and Human Services (DHHS) [29]. Victoria underwent a strict lockdown that began with restrictions imposed on July 8th 2020, followed by a declaration of a state of disaster on the 2nd of August which imposed limits on the reasons to leave one’s home, traveling only within 5 km of one’s home, a curfew from 8 pm to 5 am, and the suspension of in-person services, including schools, to mitigate the spread of the pandemic. The measures began to slowly ease on September 13 and by September 27 the lockdown began to be loosened given the falling number of daily cases. The results of the mitigation measures resulted in a successful suppression of the pandemic with regards to community transmission given that as of November 27, 2020, Victoria had recorded no new cases for 28 consecutive days [30, 31].

For the *other documents* category, we searched several government websites that provided information regarding the COVID-19 pandemic. In this case, most of the documents were sourced from the websites of the Australian Government: Department of Health (DOH), Department of Education, Skills and Employment (DESE), the National Disability Insurance Scheme (NDIS) and the Department of Social Services (DSS). Some other documents were also sourced from the state of Victoria’s Department of Health and Human Services (DHHS); because of the great number of cases in the state and the second wave of cases as well as ensuing lockdown measures that occurred between July and October of 2020. We selected documents that spoke directly to measures, guidelines, and indications to be followed by the general public during the pandemic.

### Data Analysis

We used framework analysis to examine the included sources. The steps we followed included:

- *Familiarisation*: In this step, we familiarised ourselves with relevant literature and performed a cursory read of the dataset.
- *Identifying a thematic framework*: We developed a framework based on guidelines for disability inclusion published by the International Labour Organisation [32], the United Nations Office of the High Commissioner for Human Rights [33], and the World Health Organisation [34]. Table 2 presents the adapted thematic framework we developed from the synthesis of the different recommendations.
- *Indexing*: We used the thematic framework across the two data categories, legislation and other documents. At this stage, we examined the data for information that was relevant to the thematic framework, and we made necessary adjustments to the framework, to ensure it was relevant and reflected the data. First, we broadened the focus of the analysis to include other meaningful data (what we classified as “other documents”). This other data was communicating government policy that would have otherwise been captured by looking solely at legislation - mainly because of the decision making residing in cabinet members with powers granted by declared state of emergencies. Second, as shown in Table 2, the themes were adapted to account for the sociocultural and political context of Australia and subsequently one of the themes (inclusion to decision making process), was excluded from the detailed thematic analysis. This was done because we concluded that it was more appropriate to discuss the participation of disabled people and their representative organisations in regard to the analysed data as part of the overall findings of this paper. We evaluated the data by comparing each document to the themes of the framework. This approach consisted of evaluating the definitions of each theme and assess if the document had elements that would warrant it to be classified as belonging to and/or answering the characteristics of that theme. Classification of the data to each theme relied on the content and method of access, not on the intended audience or aim of the legislation and other documents. The thematic analysis was reliant on the presence of the themes in the data.
- C*harting*: This phase focused on data extraction. We kept a spreadsheet that included the name of each data document and brief notes on each theme found in it.
- *Mapping*: Here, we examined the findings for emerging patterns that illustrated the disability inclusiveness of the policy landscape. As such, the legislation and documentation were classified as being inclusive of people with disabilities to the extent of its comparison with the thematic framework.

**Table 2:** Thematic framework

Theme	Definition
**Consideration of the needs of disabled people who face multiple exclusions**	Measures and recommendations taken to protect disabled people who are in increased risk of social exclusion and poverty, such as women, children, homeless people, prisoners, migrants/refugees, and members of Culturally and Linguistically Diverse (CALD) communities. This also includes any consideration aimed at Aboriginal and Torres Strait Islander people.
**Accessible information**	Provision of all information in accessible formats, including sign language translation, listen options, easy read. In addition, the provision of these accessible formats in languages other than English.
**Access to healthcare**	Care taken to ensure equitable access to pre-pandemic level of healthcare, including measures addressing disability-based discrimination.
**Access to education**	Measures taken to ensure remote (or limited in-person) learning is fully accessible.
**Financial support**	Provision of financial support (e.g. cash supplements or benefits), to disabled people and their family members/carers, if they had to stop working, and measures taken to ensure such access, including automatic extension of disability benefits such as disability pension and carer’s pension.
**Protection of people living in residential settings**	Measures taken to ensure people living in residential and aged care facilities are protected from infection.
**Reasonable accommodations for disabled people**	Adjustments to public health measures to accommodate the needs of disabled people, including flexibility in restrictions on movement in public spaces and other restrictions expected of the non-disabled population.

## Results

We found that most of the resources were not aimed at disabled people. The documents were written for the general, non-disabled, population. In order to find information about guidelines and practices specific to disabled people, it was necessary to visit specific websites and government agencies.

Table 3 presents an overview of the pieces of legislation that were included in the study. The full list can be seen in Additional file 1. We selected 83 legislation documents. This included new legislation introduced because of the pandemic, and amendments to existing Acts and statutory rules to include COVID-19 in their scope.

**Table 3:** COVID-19 related legislation enacted in Australia

State	Legislation register	Legislation given royal assent	Acts	Statutory rules
Commonwealth of Australia	https://www.legislation.gov.au/	13	13	0
Australian Capital Territory	https://www.legislation.act.gov.au/	14	14	0
New South Wales	https://www.legislation.nsw.gov.au/	4	4	0
Northern Territory	https://legislation.nt.gov.au/	2	2	0
Queensland	https://www.legislation.qld.gov.au/	4	4	0
Tasmania	https://www.legislation.tas.gov.au/	9	7	2
Victoria	https://www.legislation.vic.gov.au/	12	8	4
Western Australia	https://www.legislation.wa.gov.au/	9	6	3
South Australia	https://www.legislation.sa.gov.au/	16	4	12
Total		83	62	21

Table 4 presents an overview of the 114 other documents that were included in the study. The full list can be seen in Additional file 2.

**Table 4:** COVID-19 documentation published by government agency or organisation

Agency	State or federal	Total documents analysed
Department of Health	Federal	50
Department of Health and Human Services	State - Victoria	30
Department of Social Services	Federal	9
National Disability Insurance Scheme	Federal	8
Department of Education, Skills and Employment	Federal	8
Office of the Prime Minister	Federal	5
Services Australia	Federal	2
Victoria State Government	State - Victoria	1
Australian Government	Federal	1
Total		114

Of the total of 83 pieces of legislation that were analysed, 30 were specifically aimed at addressing issues that arose because of the COVID-19 pandemic. The rest can be considered amendments or minor changes to existing Acts to account for the changes that have happened because of the pandemic. All but one of the thirteen Acts passed by the Australian government were economic stimulus related; the remaining Act was related to privacy and contact information regarding the government’s mobile contact tracing application.

Out of the 114 documents classified as other documents, 54 were aimed at disabled people, their carers, and other support workers. Out of these 54 documents, 33 were written with language suggesting they were aimed at disabled people, including 7 web videos from the DSS with Australian Sign Language versions of documents and information. The remaining 21 documents were aimed at carers or people who work with disabled people. Most of these documents – 29 – were published by Australian Government agencies, while the rest were published by the DHHS, on behalf of the Victorian Government.

The remaining 60 documents that were not aimed at disabled people provided information and guidance to the general public. Because of the broad scope of these documents, disability or disabled people were rarely explicitly mentioned. The exception to this last point was the guidance for using masks in public spaces, which explicitly mentioned having a disability as a reason to be exempt from wearing a mask in public.

Finally, we noted that only five of the documents were either prepared or informed by advisory groups/bodies including ad-hoc committees and organisations such as the Council for Intellectual Disability and the Victorian Disability Advisory Council. Though, these resources were obtained from government body websites, they included contact details for organisations associated or affiliated with disabled people. One of the documents from the DOH, mentioned principles of governance and consultation with disabled people and relevant stakeholders as part of their Management and Operational Plan for People with Disabilities.

### Consideration of the needs of disabled people who face multiple exclusions

Only six pieces of legislation addressed people with multiple exclusions, yet the framing was around what the legislation called a *vulnerable person*. As such, the legislation analysed was not specific to disabled people but could apply to a disabled person. The legislation analysed addressed issues pertaining: care agreements and arrangements as well as flexibility in not being able to fully meet these arrangements given the pandemic without risk of penalty; alternative arrangements to avoid in-person attendance by people affected by family violence; expanded the definition of *vulnerable person* to include disabled people under the Public Health Emergency act of the Australian Capital Territory; information to carers of disabled people regarding contact tracing efforts; and including the COVID-19 pandemic in situations where impaired decision making falls under the rights, expectations, and responsibilities of those who have power of attorney. Five of these policies were formulated by the Australian Capital Territory, and one by the Australian Government.

Overall, we found that there were limited considerations made. Forty-one of the documents analysed fell under the purview of the theme, but only 17 of these were specifically aimed at disabled people, their carers, or support workers. One of these documents from the state of Victoria highlighted a boost in funding for advocacy and additional support for children and students, people under guardianship, disabled people in the forensic disability and youth.

Nine of the documents focused on translation and interpreting services that were available to speakers of languages other than English and Culturally and Linguistically Diverse (CALD) communities. The documents also addressed the mental health needs of young people and those in aged care given necessary isolation due to COVID-19 exposure or illness by providing resources and guidelines. Some of these publications provided contact details or redirected the reader to organisations associated or affiliated with Aboriginal and Torres Strait Islander peoples, people in aged care, international students, remote communities, trauma, family violence, and specialist schools.

### Accessible information

We found that all but one state’s legislation (Tasmania) could be exported and read in PDF format. Three of the states offered a Microsoft Word document export format (.docx), South Australia offered files in rich text format (.rtf), and only one state, Tasmania, offered an option to change the size of the font on its legislation webpage.

From the resources analysed under *other documents*, almost all were accessible in a PDF format. Of the available PDFs, the majority were offered in easy read or easy English format. Those that were not, were in a webpage format or available as a downloadable Word document. Only 37 of the total 114 of the documents did not have any type of accessibility option. Of the webpages that offered accessibility features, 41 of them had a listen option. It is important to note, however, that this option was only available in English. The web pages lacked translation features and only 25 of the documents had easy read versions. All information and documents were available in standard Australian English.

Resources with the more accessible information were the ones targeted to disabled people. However, these had to be sought out from specific agencies. As an example, the DSS offered Australian Sign Language friendly COVID-19 resources in the form of web videos. The DSS web pages also featured visual aids such as icons (e.g., an illustration of a cartoon character using a phone to represent helplines).

### Access to healthcare

Only one Act from the Australian Government addressed access to healthcare of disabled people. While the Act was allocating funding to specific services provided by the NDIS, it was not adding any further funding to the scheme because of the COVID pandemic. Twenty-four of the other documents addressed access to healthcare. These resources provided guidelines and information (including on PPE and financial schemes) aimed at healthcare professionals and support workers in the areas of mental health and disability. Limited information and resources were directly addressed to disabled people. We found that much of the disability-focused information on healthcare was available via the NDIS webpage. Some of the key focus areas of these resources were the availability of telehealth, telemedicine, personal protective equipment, and payment schemes for support workers.

There were some measures announced to support the purchase of assistive technology for eligible beneficiaries of the NDIS. This assistive technology was for the use of telehealth as well as the purchase of low-cost disability-related health consumables for those that rely on face-to-face contact with support workers. These are examples of the very few specific accommodations made available for disabled people to have access to healthcare. It must be noted that personal protective equipment (PPE) access was limited to registered NDIS providers.

One important Medicare^5^ benefit that was modified during this time, was an expansion on the limit of ten mental health sessions that are subsidised. This change doubled the total of subsidised sessions with a psychologist to twenty. This change was for all Medicare recipients, not just disabled people.

### Access to education

None of the legislation analysed addressed access to education for disabled people. Resources under other documents provided basic information on operation of schools and contact details for organisations associated or affiliated with disabled people. Only 7 documents provided limited information that was made available for students with disabilities via the DESE’s website, such as particular schools remaining open in certain locations^6^. The resources focused on students’ access to schooling as education was moved from face-to-face to remote learning. This involved a list of principles related to protective measures related to COVID-19 for schools and students to follow. The department also published recommendations for extracurricular and online socialisation activities for students and schools to utilise.

### Financial support

Only two Acts addressed financial support during the pandemic. However, the legislation briefly addressed: the payments or options for leave (as well as long service leave) for employees who work in care facilities; and the extension and renewal of professional registrations. In addition to this, there were several budgetary bills that specified a variety of economic stimulus and benefits packages to several industries and people under current financial support programs. The onset of the pandemic saw the introduction of a coronavirus supplement of AUS$550 per fortnight for people in eligible support income payments until September 2021; then the payment was reduced to AUS$250 until December 2020 [36]. The pandemic also saw the re-branding of the previous unemployment benefit to a new program called JobSeeker (formerly Newstart) and with it an increase in fortnightly payments that amounted to double of what it was before until September 2020 [37, 38]^7^. The federal government also introduced a wage subsidy program aimed at employers called JobKeeper, where employers could continue paying their employees where it otherwise would not have been possible, because of the economic impact of the pandemic on their business [39]. Beneficiaries of DSP and Carer Payments were excluded from these specific coronavirus supplements [40]. While any person with a disability employed by a business that receives JobKeeper, will receive the JobKeeper payments, such payments are counted as additional income and might affect the beneficiary’s DSP [40].

Only 8 documents addressed financial support regarding disabled people. However, we found that most of the information regarding financial support was largely available for (and aimed at) carers of disabled people. This included access and/ or changes to carers’ payments in relation to the JobSeeker and JobKeeper programs. The Victorian government committed additional funding ensuring accessibility and appropriate linkages with health and disability supports at each coronavirus assessment centre.

### Protection of people living in residential settings

Only two legislative documents addressed the needs of people living in residential settings, and one of them was not specific to disabled people. South Australia passed regulations that specified several accommodations that housed and supported disabled people, mental health inpatients, and aged care would fall under their protected person definition. In contrast, the Act from the Australian Capital Territory addressed the changes needed to hold meetings without the in-person attendance of the residents. Twenty-three documents addressed the theme of people living in residential settings. Resources under other documents indicated the key measures taken to protect people living in residential settings were aimed at organisational levels. The Department of Health provided specific guidelines and recommendations in place for organisations providing residential care and their employees. Fact sheets and guidelines for supported residential service proprietors and their staff provided guidelines for the appropriate accommodations that had to be made in relation to COVID-19 and the people in residential care. There were some general recommendations such as information that directed people to existing schemes and support programs. On the other hand, there was tailored and specific information that directed the use of face masks, guidelines for the isolation of individuals suspected or suffering from COVID-19, information on priority testing, among other recommendations. Although disabled people were recognised in some of these documents as a group at greater risk of contagion, only a minority of the documents were aimed at this population.

### Reasonable accommodations for disabled people

Of the legislation analysed, only two Acts directly addressed reasonable accommodations towards disabled people. The two Acts passed by the Australian Capital Territory addressed voting participation by disabled people in the upcoming territory’s election through electronic means or by phone. The other change pertained to updating decision making regarding medical treatment by a trustee or guardian under the territory’s Mental Health Act. An additional eight legislative documents did not directly address disabled people, but their scope did affect this population. Four of these legislation documents waived in-person requirements of various issues and were substituted by alternative methods; two pertained to rent relief and breaches of contracts during the pandemic; one expanded the definition of an ‘ill’ person to a more comprehensive definition; and one was related to police dealing with a person from a protected category and intentional transmission of the COVID-19 virus.

Thirty-one resources under other documents addressed the issue of reasonable accommodations for disabled people. In particular, the resources highlighted some key accommodations made for disabled people including targeted information with the use of inclusive language and communications, details regarding the expansion of resources available to disabled people, and guidelines and communications aimed at support workers and other healthcare providers.

Out of the 31 resources analysed, 17 were targeted at disabled people, support workers, and carers. One of the key messages of the documents was the state of Victoria’s guidelines which exempted disabled people from the mandated use of facemasks outside of their homes. The messages from the DHHS analysed under this theme included the use of images of a diverse range of people that included disabled people. The documents outlined some guidelines for practitioners and support workers that work with people classified as high-risk of infection, which included disabled people. Further, almost every publication from various agencies provides contact information for disability support services.

## Discussion

The findings indicate a large number of legislation, policies, and other documents outlining the response to COVID-19, at the state and Australian Government levels. However, despite this seemingly comprehensive response, the needs of disabled people were not fully addressed. Furthermore, the structure of the Australian political system means that there are disparities across the different states, with each implementing its own policies in areas such as education, delivery of health care services, among others.

The findings revealed the existence of a few direct and several indirect measures. Direct measures were those that were drawn with specific reference to disabled people, while indirect measures were those that also affected disabled people, along with other groups of the population. Disability-inclusiveness appeared to be inconsistent and not fully considered, leaving disabled people exposed to greater risk of COVID-19, with the exception of South Australia that ensured that disabled people living in residential settings were included in their definition of a protected person. A failure of governments to fully protect disabled people against reduced access to or exclusion from healthcare, employment, and social care, and the lack of explicit measures taken to address instances of medical rationing, will inevitably lead to exacerbating existing inequities, further disadvantaging disabled people [41].

Neoliberal policies in the health and welfare sector in Australia [42] and elsewhere [43] have led to an individualisation of the responsibility to remain healthy and a reliance on people as autonomous and independent consumers. People are being blamed, and sometimes penalised, for poor health outcomes with little or no consideration of structural factors. This personalisation rhetoric [42] transforms disabled people into liabilities who use up limited resources, and they are directly or indirectly excluded from the healthcare system, as examples from countries that have a long history in implementing neoliberal policies demonstrate [44]. Yet, there are strategies available to promote greater inclusion – and thus health equity – for all structurally vulnerable peoples. The move to a Health in All Policies (HiAP) approach is one such strategy [16], yet has only been implemented in one Australian state (South Australia, 17). However, the influence of this initiative was unclear in our analysis.

The intersections of *ubiquitous disablism* [45] with a discourse of individualised responsibility in the face of COVID-19 protection, put at risk disabled people. The failure of states across the world to provide care to those most at need, is exemplified through shifting the responsibility to individual actors (and also the blame for non-compliance), with little acknowledgment of structural factors that intersect with infection and contagion. Little, if any, consideration is given to the fact that, as Kochhar argues, while disabled people are asked to shield and protect themselves, “most of this discourse is able-bodied — it concerns bodies *capable* of these “essential measures” in the first place” [46]. As Andrews et al. warn, disablism and its associated “message that some lives are more worthy than others” is already being translated into policy [47]. This is more explicitly exemplified in medical rationing practices and their justification [45] which are often based on a neo-eugenics discourse, decreeing some bodies as expendable.

## Conclusions

This article adds to a growing body of evidence showing how disabled people experience structural vulnerabilities, which are exacerbated during the COVID-19 pandemic. Governments need to take a clear stance towards the emergence of such a discourse that actively disvalues disabled people. If one thing has become clearly evident, is that the idea of an “autonomous individual body” [46] is a fiction that affects everybody, not just disabled people: we are connected through risk and vulnerabilities that travel between bodies.

## Supplementary Information

**Additional file 1.** Legislation analysed.

**Additional file 2.** Other documents analysed.

## Data Availability

The datasets used and/or analysed during the current study are available from the corresponding author on request. Additional files 1 and 2 provide the names of the legislation and documents that were used to generate our dataset.

## Abbreviations

CALD: Culturally and Linguistically Diverse
CRPD: Convention for the Rights of People with Disabilities
DESE: Department of Education, Skills and Employment
DHHS: Department of Health and Human Services, State Government of Victoria
DOH: Australian Government Department of Health
DSP: Disability Support Pension
DSS: Australian Government Department of Social Services
HiAP: Health in All Policies
NDIS: National Disability Insurance Scheme
PPE: Personal protective equipment

## Footnotes

1 Sociologist Deborah Lupton [24] has developed a comprehensive timeline of COVID-19 in Australia; see https://deborahalupton.medium.com/timeline-of-covid-19-in-australia-1f7df6ca5f23.

2 Also called the ‘Disability Royal Commission’, this is a major independent public inquiry into the historical and contemporary treatment of disabled people in Australian society. More information can be found at: www.disability.royalcommission.gov.au.

3 For more information on the NDIS eligibility process, please see: Am I eligible | NDIS.

4 Information on DSP eligibility and payments can be found at: Disability Support Pension - Services Australia.

5 A mental health treatment plan is a Medicare benefit that allows a general practitioner to refer their client to a mental health care professional. Medicare will either cover the cost or partially subsidise (depending on the fees set by the mental health professional) a set number of visits for this treatment. Before the pandemic, the limit of subsidised visits was ten; they are currently capped at twenty given the circumstances of the pandemic [35].

6 In Australia, state governments have authority regarding the education sector, and they are responsible for guidelines for school, universities, and other educational institutions.

7 It then later underwent some changes and further extended until March 2021. The initial payment was halved, and then supplemented with a minor increase.
