# Supplementary material for: Examining the role of governmsent in shaping disability inclusiveness around COVID-19: a framework analysis of Australian guidelines
Source: Int J Equity Health. 2021 Jul 16;20:166. doi: 10.1186/s12939-021-01506-2 (PMC8283747; doi:10.1186/s12939-021-01506-2)
Supplement: Supplementary file 1 — Additional file 1. Legislation analysed. [file 12939_2021_1506_MOESM1_ESM.docx]

**Additional file 1: Legislation analysed**

| **Type** | **Government** | **Name** |
| --- | --- | --- |
| Act | Federal | Treasury Laws Amendment (2020 Measures No. 3) Bill 2020 |
| Act | Federal | Privacy Amendment (Public Health Contact Information) Bill 2020 |
| Act | Federal | Appropriation Bill (No. 5) 2019-2020 |
| Act | Federal | Appropriation Bill (No. 6) 2019-2020 |
| Act | Federal | Coronavirus Economic Response Package (Payments and Benefits) Bill 2020 |
| Act | Federal | Appropriation (Coronavirus Economic Response Package) Bill (No. 1) 2019-2020 |
| Act | Federal | Appropriation (Coronavirus Economic Response Package) Bill (No. 2) 2019-2020 |
| Act | Federal | Assistance for Severely Affected Regions (Special Appropriation) (Coronavirus Economic Response Package) Bill 2020 |
| Act | Federal | Australian Business Growth Fund (Coronavirus Economic Response Package) Bill 2020 |
| Act | Federal | Boosting Cash Flow for Employers (Coronavirus Economic Response Package) Bill 2020 |
| Act | Federal | Coronavirus Economic Response Package Omnibus Bill 2020 |
| Act | Federal | Structured Finance Support (Coronavirus Economic Response Package) Bill 2020 |
| Act | Federal | Coronavirus Economic Response Package Omnibus (Measures No. 2) Bill 2020 |
| Act | ACT | Children and Young People Act 2008 |
| Act | ACT | Long Service Leave (Portable Schemes) Act 2009 |
| Act | ACT | Powers of Attorney Act 2006 |
| Act | ACT | Retirement Villages Act 2012 |
| Act | ACT | Working with Vulnerable People (Background Checking) Act 2011 |
| Act | ACT | Taxation Administration Act 1999 |
| Act | ACT | Education Act 2004 |
| Act | ACT | Family Violence Act 2016 |
| Act | ACT | Public Health (Emergencies)Amendment Act 2020 |
| Act | ACT | Public Health Act 1997 |
| Act | ACT | Public Trustee and Guardian Act 1985 |
| Act | ACT | COVID-19 Emergency Response Act 2020 |
| Act | ACT | COVID-19 Emergency Response Legislation Amendment Act 2020 |
| Act | ACT | COVID-19 Emergency Response Legislation Amendment Act 2020 (No 2) |
| Act | NSW | COVID-19 Legislation Amendment (Emergency Measures) Bill 2020 |
| Act | NSW | COVID-19 Legislation Amendment (Emergency Measures—Attorney General) Bill 2020 |
| Act | NSW | COVID-19 Legislation Amendment (Emergency Measures—Miscellaneous) Bill 2020 |
| Act | NSW | COVID-19 Legislation Amendment (Emergency Measures—Treasurer) Bill 2020 |
| Act | NT | PUBLIC AND ENVIRONMENTAL HEALTH ACT 2011 |
| Act | NT | RESIDENTIAL TENANCIES ACT 1999 |
| Act | QLD | Public Health (Declared Public Health Emergencies) Amendment Bill 2020 |
| Act | QLD | Public Health and Other Legislation (Public Health Emergency) Amendment Bill 2020 |
| Act | QLD | Appropriation (COVID-19) Bill 2020 |
| Act | QLD | COVID-19 Emergency Response Bill 2020 |
| SR | TAS | State Service Regulations 2011 |
| Act | TAS | Emergency Management Act 2006 |
| Act | TAS | Public Health Act 1997 |
| Act | TAS | Residential Tenancy Act 1997 |
| Act | TAS | Residential Tenancy (Shorter Period) Notice 2020 |
| SR | TAS | Payroll Tax (Pandemic) Order 2020 |
| Act | TAS | COVID-19 Disease Emergency (Commercial Leases) Act 2020 |
| Act | TAS | COVID-19 Disease Emergency (Miscellaneous Provisions) Act 2020 |
| Act | TAS | COVID-19 Disease Emergency (Miscellaneous Provisions) Act (No. 2) 2020 |
| SR | VIC | Public Health and Wellbeing Regulations 2019 |
| Act | VIC | Safe Patient Care (Nurse to Patient and Midwife to Patient Ratios) Act 2015 |
| Act | VIC | Residential Tenancies Act 1997 |
| Act | VIC | Children, Youth and Families Act 2005 |
| Act | VIC | Family Violence Protection Act 2008 |
| Act | VIC | Education and Training Reform Act 2006 |
| Act | VIC | Accident Compensation Act 1985 |
| Act | VIC | Workplace Injury Rehabilitation and Compensation Act 2013 |
| SR | VIC | COVID-19 Omnibus (Emergency Measures) (Integrity Entities) Regulations 2020 |
| SR | VIC | Residential Tenancies (COVID-19 Emergency Measures) Regulations 2020 |
| SR | VIC | COVID-19 Omnibus (Emergency Measures) (Commercial Leases and Licences) Regulations 2020 |
| Act | VIC | COVID-19 Omnibus (Emergency Measures) Act 2020 |
| SR | WA | Occupational Safety and Health Regulations 1996 |
| SR | WA | Taxation Administration Regulations 2003 |
| Act | WA | Emergency Management Act 2005 |
| Act | WA | Mandatory Testing (Infectious Diseases) Act 2014 |
| Act | WA | Residential Tenancies Act 1987 |
| SR | WA | Mandatory Testing (Infectious Diseases) Regulations 2020 |
| Act | WA | Commercial Tenancies (COVID-19 Response) Act 2020 |
| Act | WA | Pay-roll Tax Relief (COVID-19 Response) Act 2020 |
| Act | WA | Residential Tenancies (COVID-19 Response) Act 2020 |
| SR | SA | Taxation Administration Regulations 2017 |
| SR | SA | South Australian Public Health (Notifiable and Controlled Notifiable Conditions) Regulations 2012 |
| SR | SA | Legislation Revision and Publication Regulations 2017 |
| SR | SA | Gas Regulations 2012 |
| SR | SA | Electricity (General) Regulations 2012 |
| SR | SA | COVID-19 Emergency Response (Section 17) Regulations 2020 |
| SR | SA | COVID-19 Emergency Response (Section 16) Regulations 2020 |
| SR | SA | COVID-19 Emergency Response (Section 14) Regulations 2020 |
| SR | SA | COVID-19 Emergency Response (Schedule 1) Regulations 2020 |
| SR | SA | COVID-19 Emergency Response (General) Regulations 2020 |
| SR | SA | COVID-19 Emergency Response (Commercial Leases No 2) Regulations 2020 |
| Act | SA | COVID-19 Emergency Response Act 2020 |
| Act | SA | Training and Skills Development Act 2008 |
| SR | SA | South Australian Public Health Act 2011 |
| Act | SA | Payroll Tax Act 2009 |
| Act | SA | Emergency Management Act 2004 |
