# Supplementary material for: Examining the role of governmsent in shaping disability inclusiveness around COVID-19: a framework analysis of Australian guidelines
Source: Int J Equity Health. 2021 Jul 16;20:166. doi: 10.1186/s12939-021-01506-2 (PMC8283747; doi:10.1186/s12939-021-01506-2)
Supplement: Supplementary file 2 — Additional file 2. Other documents analysed. [file 12939_2021_1506_MOESM2_ESM.docx]

**Additional file 2: Other documents analysed**

| State | Type | Agency | Title of Document |
| --- | --- | --- | --- |
| Federal | News | Services Australia | How changes to JobKeeper payment may affect you |
| Federal | News | Services Australia | How Coronavirus Supplement and eligibility for some income support payments are changing |
| Federal | Media Release | Prime Minister | $1.1 BILLION TO SUPPORT MORE MENTAL HEALTH, MEDICARE AND DOMESTIC VIOLENCE SERVICES |
| Federal | Media Release | Prime Minister | FURTHER STATEMENT ON HAIRDRESSERS, BARBERS AND FUNERALS FROM NATIONAL CABINET |
| Federal | Media Release | Prime Minister | UPDATE ON CORONAVIRUS MEASURES |
| Federal | Media Release | Prime Minister | UPDATE ON CORONAVIRUS MEASURES |
| Federal | Media Release | Prime Minister | UPDATE ON CORONAVIRUS MEASURES |
| Federal | Media Release | Department of Health | $3 million to boost the national coordinated COVID-19 research response |
| Federal | Media Release | Department of Health | Additional COVID-19 Mental Health Support |
| Federal | Media Release | Department of Health | COMMONWEALTH and STATES SIGN $131 BILLION FIVE YEAR HOSPITALS AGREEMENT |
| Federal | Publication | Department of Health | Coronavirus (COVID-19) – Are cloth face masks likely to provide protection against COVID-19? |
| Federal | Publication | Department of Health | Coronavirus (COVID-19) – Help for finances and mental health |
| Federal | Publication | Department of Health | Coronavirus (COVID-19) – Information for Families |
| Federal | Publication | Department of Health | Coronavirus (COVID-19) – Information for permanent aged care residents – emergency leave |
| Federal | Publication | Department of Health | Coronavirus (COVID-19) – It’s ok to have home care |
| Federal | Guideline | Department of Health | Coronavirus (COVID-19) action plan |
| Federal | Publication | Department of Health | Coronavirus (COVID-19) advice for retirement villages |
| Federal | Publication | Department of Health | Coronavirus (COVID-19) and children |
| Federal | Webpage | Department of Health | Coronavirus (COVID-19) health alert |
| Federal | Publication | Department of Health | Coronavirus (COVID-19) in Australia – Pandemic Health Intelligence Plan |
| Federal | Publication | Department of Health | Coronavirus (COVID-19) information for employers |
| Federal | Publication | Department of Health | Coronavirus (COVID-19) information for older Australians |
| Federal | Publication | Department of Health | Coronavirus (COVID-19) Living well in the COVID-19 pandemic |
| Federal | Website | Department of Health | Coronavirus (COVID-19) resources for the general public |
| Federal | Website | Department of Health | Coronavirus (COVID-19): National Health Plan resources |
| Federal | Publication | Department of Health | COVID-19 and the Commonwealth Home Support Programme – information for clients, families and carers |
| Federal | Publication | Department of Health | COVID-19 National Health Plan – prescriptions via telehealth – a guide for patients |
| Federal | Webpage | Department of Health | COVIDSafe app |
| Federal | Publication | Department of Health | Flu vaccination requirements for aged care facilities |
| Federal | Presentation | Department of Health | Impact of COVID-19 in Australia – ensuring the health system can respond |
| Federal | Website | Department of Health | Main Website - [health.gov.au/covid-19](http://health.gov.au/covid-19) |
| Federal | Publication | Department of Health | Management and Operational Plan for People with Disability |
| Federal | Media Release | Department of Health | Strengthened guidelines to protect aged care residents |
| Federal | Publication | Department of Health | When should masks be worn in the community, in Australia? |
| Federal | Webpage | Department of Education, Skills and Employment | Coronavirus (COVID-19) information for job seekers |
| Federal | Webpage | Department of Education, Skills and Employment | Coronavirus (COVID-19) information for schools and students |
| Federal | Media Release | Department of Education, Skills and Employment | Early Childhood Education and Care Coronavirus (COVID-19) Information |
| Federal | Media Release | Department of Education, Skills and Employment | Higher education relief package - Frequently Asked Questions |
| Federal | News | Department of Education, Skills and Employment | JobTrainer package announced |
| Federal | Webpage | Australian Government | Advice about COVID-19 coronavirus |
| Federal | Media Release | National Disability Insurance Scheme | Delivering the NDIS during COVID-19: Vic, NSW participants able to claim for PPE |
| Federal | Media Release | National Disability Insurance Scheme | Further initiatives to support NDIS participants and providers during coronavirus pandemic |
| Federal | Publication | National Disability Insurance Scheme | NDIS COVID-19 response – Victoria |
| Federal | Media Release | National Disability Insurance Scheme | NDIS update for Victoria |
| Federal | Media Release | National Disability Insurance Scheme | New measures to support NDIS participants and providers through COVID-19 |
| Federal | Media Release | National Disability Insurance Scheme | Two million more face masks for Victorian aged care and disability workers |
| Federal | Media Release | National Disability Insurance Scheme | Update on NDIS coronavirus response |
| Federal | Media Release | National Disability Insurance Scheme | Vic and NSW Providers to directly claim costs of PPE |
| Federal | Publication | Department of Social Services | Auslan version of Coronavirus (COVID-19) accessible resources |
| Federal | Publication | Department of Social Services | Auslan version of Do you want to make a complaint |
| Federal | Publication | Department of Social Services | Auslan version of Helping others |
| Federal | Publication | Department of Social Services | Auslan version of Looking after yourself |
| Federal | Publication | Department of Social Services | Auslan version of Support for your situation |
| Federal | Publication | Department of Social Services | Auslan version of the Disability Information Helpline |
| Federal | Publication | Department of Social Services | Auslan version of the Who else can you talk to? |
| Federal | Publication | Department of Social Services | Fact sheet - JobKeeper Payment impacts on Carer Payment Recipients - Easy Read |
| Federal | Publication | Department of Social Services | Fact sheet - JobKeeper Payment impacts on Disability Support Pension Recipients - Easy Read |
| Federal | Publication | Department of Health | About the COVIDSafe app – Easy Read |
| Federal | Website | Department of Health | Accessing health services during coronavirus (COVID-19) restrictions |
| Federal | Publication | Department of Health | Coronavirus (COVID-19) Guide for Home Care Providers |
| Federal | Publication | Department of Health | Coronavirus (COVID-19) hospital companion for people with disability |
| Federal | Publication | Department of Health | Coronavirus (COVID-19): Our plan for keeping people with disability safe – Easy Read |
| Federal | Publication | Department of Health | Coronavirus: 5 things to do right now – Easy Read |
| Federal | Publication | Department of Health | Coronavirus: frequently asked questions – Easy Read |
| Federal | Publication | Department of Health | Coronavirus: Information for people with disability and their supporters – Easy Read |
| Federal | Publication | Department of Health | Coronavirus: Social distancing – Easy Read |
| Federal | Publication | Department of Health | Coronavirus: Staying at home – Easy Read |
| Federal | Publication | Department of Health | Coronavirus: Wearing a mask – Easy Read |
| Federal | Publication | Department of Health | Coronavirus: What is it? – Easy Read |
| Federal | Publication | Department of Health | Coronavirus: What you need to know – Easy Read |
| Federal | Publication | Department of Health | Coronavirus: Who can you call? |
| Federal | Publication | Department of Health | Guide to personal protective equipment (PPE) for disability care providers |
| Federal | Publication | Department of Health | If you think you have coronavirus – Easy Read |
| Federal | Publication | Department of Health | Information for Disability Support Providers and Workers |
| Federal | Publication | Department of Health | Information for health and mental health workers supporting people with disability |
| Federal | Publication | Department of Health | Information for primary health care services supporting people with disability |
| Federal | Publication | Department of Health | Information for support workers and carers on coronavirus (COVID-19) testing for people with disability |
| Federal | Publication | Department of Health | Looking after your health during coronavirus – Easy Read |
| Federal | Publication | Department of Health | Return to School for Students with Disability COVID-19 Health Risk Management Plan |
| Federal | Publication | Department of Health | Your emergency plan for COVID-19 – Easy Read |
| Federal | Website | Department of Education, Skills and Employment | COVID 19 Resources for teachers and school leaders |
| Federal | Website | Department of Education, Skills and Employment | COVID-19 National Principles for School Education |
| Federal | Website | Department of Education, Skills and Employment | COVID-19 Support for students with disability |
| Victoria | Media Release | Victorian Government | Coronavirus (COVID-19) restrictions Victoria |
| Victoria | Publication | Department of Health and Human Services | Mental health and coronavirus (COVID-19) information for those in isolation (Word) |
| Victoria | Publication | Department of Health and Human Services | Coronavirus - emergency relief packages - easy read version (PDF) |
| Victoria | Publication | Department of Health and Human Services | Coronavirus - looking after your mental health - easy read version (PDF) |
| Victoria | Publication | Department of Health and Human Services | Coronavirus (COVID-19) Information for people with disability and their carers while in self-isolation (Word) |
| Victoria | Publication | Department of Health and Human Services | Coronavirus (COVID-19) Isolation management in disability accommodation (Word) |
| Victoria | Publication | Department of Health and Human Services | Coronavirus (COVID-19) Plan for the disability services sector - Version 7 - 12 July 2020 (Word) |
| Victoria | Publication | Department of Health and Human Services | Coronavirus (COVID-19) resources for people with disability (Word) |
| Victoria | Publication | Department of Health and Human Services | Coronavirus (COVID-19) restrictions - information for disability service providers (Word) |
| Victoria | Publication | Department of Health and Human Services | Coronavirus (COVID-19): Changes to disability supports and services (Word) |
| Victoria | Publication | Department of Health and Human Services | Current restrictions - if you don't live in metropolitan Melbourne and Mitchell Shire (Word) |
| Victoria | Publication | Department of Health and Human Services | Current restrictions - if you live in metropolitan Melbourne and Mitchell Shire (Word) |
| Victoria | Publication | Department of Health and Human Services | Diffusing tense situations during the coronavirus (COVID-19) restrictions (Word) |
| Victoria | Publication | Department of Health and Human Services | Do I have to wear a face mask? - Easy English - Handout (PDF) |
| Victoria | Media Release | Department of Health and Human Services | Face coverings - 11.59pm Wednesday 22 July |
| Victoria | Publication | Department of Health and Human Services | Face coverings: Frequently asked questions for people with disability and their support workers (Word) |
| Victoria | Publication | Department of Health and Human Services | Face masks at work: frequently asked questions for disability support workers (COVID-19) 22 July 2020 (Word) |
| Victoria | Publication | Department of Health and Human Services | Fact sheet on Stay at home and isolation directions for Supported Residential Services proprietors and staff (Word) |
| Victoria | Publication | Department of Health and Human Services | Factsheet on Care facilities - Disability accommodation supplementary guidance (Word) |
| Victoria | Publication | Department of Health and Human Services | Family violence - how to get support during coronavirus (COVID-19) - easy read version (PDF) |
| Victoria | Publication | Department of Health and Human Services | Financial assistance for people with disability during coronavirus (COVID-19) (Word) |
| Victoria | Publication | Department of Health and Human Services | Mental health and coronavirus (COVID-19) information for parents and families (Word) |
| Victoria | Publication | Department of Health and Human Services | Mental health and coronavirus (COVID-19) information for seniors (Word) |
| Victoria | Publication | Department of Health and Human Services | Other proactive strategies to lessen impact of coronavirus (COVID-19) restrictions (Word) |
| Victoria | Publication | Department of Health and Human Services | People with disability and their carers - General health and wellbeing for home isolation (Word) |
| Victoria | Publication | Department of Health and Human Services | Permitted worker scheme (COVID-19) - fact sheet for disability service providers (Word) |
| Victoria | Publication | Department of Health and Human Services | Practice on a page: Telehealth and Functional Behaviour Assessments (Word) |
| Victoria | Publication | Department of Health and Human Services | Priority processing of coronavirus (COVID-19) tests for disability residential services (Word) |
| Victoria | Publication | Department of Health and Human Services | Supporting Victorians with disability during coronavirus (COVID-19) (Word) |
| Victoria | Publication | Department of Health and Human Services | Using environmental changes to lessen the impact of coronavirus (COVID-19) (Word) |
| Victoria | Publication | Department of Health and Human Services | What to expect when you get disability services – Easy read information for people with disability (Word) |
